# Strengthening social skills: developing a social competence intervention for physical education using intervention mapping—protocol paper

**DOI:** 10.3389/fpsyg.2025.1484943

**Published:** 2025-02-18

**Authors:** Iris Schüller, V. Vanessa Wergin, Filip Mess

**Affiliations:** ^1^Sport and Health Didactics, Department Health and Sport Sciences, TUM School of Medicine and Health, Technical University of Munich, Munich, Germany; ^2^Faculty of Health and Behavioural Sciences, School of Human Movement and Nutrition Sciences, The University of Queensland, Brisbane, QLD, Australia

**Keywords:** social competence, connectedness, personality development, multilevel assessment, implementation, physical education

## Abstract

**Introduction:**

The promotion of social competences using multi-method approaches is an understudied area in education and psychology. This study addresses the gap by developing and presenting a program to enhance social competences using theory-derived program and assessment designs.

**Materials and equipment:**

Bartholomew’s Intervention Mapping (IM) approach, initially used for health promotion, was innovatively applied to a psychological and educational context. The development process and implementation challenges are presented in this article.

**Methods:**

The six-step IM process was adapted to create a comprehensive program design that models social behavior, specifically for Physical Education in schools. The program targets perceptive-cognitive, emotional-motivational, and behavioral components of social competence, along the social competence model by Kanning. Results on effect sizes have yet to be calculated.

**Discussion:**

The IM process was time-consuming and extensive but provided a systematic structure, methodological quality, and traceability of effects. Future adaptations of this program could include extensions to different situational contexts and specific need groups, such as classes with a history of bullying or psychological conditions. This study contributes valuable insights into using the IM approach for promoting behavioral change in a systematic and evidence-based manner.

## 1 Introduction

### 1.1 Importance of the research topic

Social competence is essential for children to communicate effectively, develop healthy relationships, and navigate social challenges during childhood, adolescence, and adulthood (Junge et al., 2020). Schools play a crucial role in fostering these skills by providing structured environments where students can learn cooperation, empathy, and conflict resolution (Collie, 2020). Teaching social competence promotes emotional well-being, reduces bullying, and enhances academic success by creating inclusive and supportive classroom dynamics (Sancassiani et al., 2015).

The promotion of social competences offers opportunities to prevent risk developments in children and adolescents like drug abuse, attention deficits, aggression, violence and bullying (Griffin et al., 2001, pp. 485–498; Scheithauer and Bull, 2010, pp. 266–281; Smokowski et al., 2004, pp. 233–251; Thorell and Rydell, 2008, pp. 584–595). Furthermore, students with high levels of social competences tend to be more successful academically and in their professional career (Scheithauer et al., 2010, pp. 54–68). Moreover, social competence is described as part of the health-related quality of life and satisfying social relations as a life-long need (Karasimopoulou et al., 2012, pp. 780–793; Pinquart and Sorensen, 2000, pp. 187–224). Finally, students with high social competence levels were also found to be more popular within their peer group and influence the school climate positively (Gulay et al., 2010, pp. 77–92; Jerusalem and Klein-Heßling, 2002, pp. 164–174).

### 1.2 Legitimization for an intervention in school settings and PE

Due to the complexity of social interactions, the construct of social competence is the “product of a wide range of cognitive abilities, emotional processes, behavioral skills, social awareness, and personal and cultural values related to interpersonal relationships” (Orpinas, 2010, pp. 1–2). The school setting has since been identified as an ideal setting for the promotion of social competences allowing it to systematically and institutionally address children of different socio-economic backgrounds within stable peer groups (Goldberg et al., 2019, pp. 755–782; Noel et al., 2009).

On a political level, legitimization can be found in the statements of the World Health Organization (2007), which states that social interactions should also be considered as an important part of physical activity and the development of social values and skills in children and young people. On an organizational level, Physical Education allows to address physical activity and these social aims in an institutional setting. Nevertheless, only two studies focused on promoting physical activity, when aiming at an improvement of social competences (Bundy et al., 2011, p. 680; Polvi and Telama, 2000, pp. 105–115) and did not report detailed results.

### 1.3 Existing research and research gap

Sports and Physical Education (PE) have been shown to be effective tools for the development of skills and competences in youth (e.g., Fraser-Thomas et al., 2005, pp. 19–40; Gould and Carson, 2008, pp. 58–78; Holt et al., 2011, pp. 490–499; Weiss, 2011, pp. 55–65). However, participation in sport and PE alone does not automatically create positive outcomes (e.g., Cryan and Martinek, 2017, pp. 127–149; Fraser-Thomas and Côté, 2009, pp. 3–23). Instead, PE teachers and sport coaches need to create an environment allowing for the development of social competence (Opstoel et al., 2020, pp. 797–813). To promote social competences successfully, interventions are most effective during sensitive development stages (Rose-Krasnor, 1997, pp. 111–135) and should be systematically derived from theoretical constructs and focus on individual strengths rather than on problems (Benson et al., 2006, pp. 894–941).

Systematic reviews on studies promoting social competence in schools within PE or the context of physical activity suggest, that current intervention programs lack a systematic derivation of program content along with clearly defined strategies, goals, assessment methods, and evaluation plans based on a theoretical background or construct, which is mostly because definitions and aspects vary widely (Opstoel et al., 2020, pp. 797–813; Schüller and Demetriou, 2018, pp. 39–55).

It is necessary to implement multi-level assessment tools including self-assessment, dependent and independent observations, as well as network analyses to cover all aspects of the multi-dimensional construct of social competences (Schüller and Demetriou, 2018, pp. 39–55). Variables like duration and frequency do not seem to influence program effects, but many studies show a lack of methodological quality (Opstoel et al., 2020, pp. 797–813; Schüller and Demetriou, 2018, pp. 39–55). A lack of systematic intervention and implementation planning thus excludes the option of a target-oriented classification of correlations and effects (Lloyd et al., 2011, pp. 1–15).

Consequently, it is essential for interventions to (a) increase methodological quality in the study design, (b) derive program contents and assessment strategies from theoretical constructs, and (c) develop conclusive models for intervention aspects and multi-level assessment plans (Schüller and Demetriou, 2018, pp. 39–55).

### 1.4 The intervention mapping protocol as a tool

Systematic intervention planning is thus a key element for the development and effective implementation of intervention programs for target groups. The six step process of the IM Protocol is therefore defined by the keywords “planning, research, and theory” (Bartholomew Eldrege et al., 2016). It has been refined multiple times since its first publication in 1998 (Kok et al., 2017, p. 19) and has been applied to a variety of contexts in studies promoting health related objectives since 2008 (Majid et al., 2018), targeting implementations not only toward patients and a clinical setting, but also in environmental and political contexts (Fernandez et al., 2019b, p. 158).

An advantage of utilizing IM is the structure the IM process offers along the line of an expert driven set of guidelines, which is especially important in a growing research field - like the promotion of social competences - because it only includes few large scale, and long term studies (Schüller and Demetriou, 2018, pp. 39–55). Therefore, following a targeted approach in intervention planning makes it possible to base intervention programs on statistically and methodological relevant considerations and standards (Godin et al., 2007, pp. 138–142). Stakeholder involvement allows for a higher level of legitimation and acceptance of program contents (Majid et al., 2018).

### 1.5 Objective and novelty of the study protocol

The objective of the current study protocol is to promote social competence in PE by developing a systematically derived, evidence-based intervention program that can be applied in Physical Education in schools. The program design is based on perceptive-cognitive, emotional-motivational, and behavioral components of Kanning (2002, pp. 154–163) theory on social competences and describes the IM process supporting the planning and development of the intervention design including the assessment and evaluation planning.

To accomplish this, the study aims to (a) develop a systematic derivation, evidence-based intervention program for the promotion of social competence in PE; (b) use the principles of Intervention Mapping (IM) protocol to ensure a structured, theory-driven approach to program development; (c) include multi-level-assessment instruments in a more comprehensive evaluation of the multidimensional construct of social competence.

The novelty of this study lies in the rigorous application of the IM process to the context of social competence promotion in PE. Such an approach can yield a more effective targeting and designing of interventions that close the gap between theory and practice. The next section will give a general overview over the intervention mapping process as it is intended by Bartholomew Eldrege et al. (2016). Afterwards, the application toward the promotion of social competence in PE is presented in the results section. Finally, the discussion will pick up the challenges, limitations and suggestions derived from an application of each of the intervention mapping steps toward the promotion of social competence.

## 2 Materials and equipment

### 2.1 IM approach

The intervention program promoting social competence was dependent on a few context-specific factors, which included (a) the setting of PE in school, (b) the delivery of the intervention content by teachers, (c) the students’ age of 12–14 years to fit the adolescent development stage, and (d) the curriculum specific demands for PE lessons. Additionally, research suggests, that a theory-derived intervention program and assessment plan including a multi-level approach promoting different aspects of social competence, and a multi-method assessment strategy using a variety of tools would be beneficial (Schüller and Demetriou, 2018, pp. 39–55). The intervention mapping, which is described in the following sections, was then fitted and tailored along these predetermined factors.

The IM approach by Bartholomew Eldrege et al. (2016) is used to systematically plan intervention programs and consists of six main steps that are iteratively executed. These steps include (1) the creation of a logic model and a description of the problem approached, (2) the definition of targeted program outcomes deriving of a logic model of change, (3) a detailed scope of the program design, (4) the preparation of steps necessary for the program production including a content schedule, (5) a program implementation plan, and (6) an assessment and evaluation plan (Bartholomew Eldrege et al., 2016). The aim of a development based on IM is to explicitly focus on the objectives in relation to the target population and its characteristics (Bartholomew Eldrege et al., 2016). The fundament of IM is combining behavioral theory and evidence from existing research to develop overall behavioral objectives and determinants as well as learning objectives and determinants (Bartholomew Eldrege et al., 2016). Furthermore, programs need to be tailored to the targeted population and the internal and external determinants associated (Lloyd et al., 2011, pp. 1–15).

#### 2.1.1 Application of step 1: logic model of the problem—needs assessment

The first step of the IM approach aims at the development of a logic model of the problem including a needs assessment or detailed analysis of the problem and is concluded with a definition of possible outcomes.

The process started with the selection of adaptable and applicable theoretical construct of social competences. The wide variability of definitions, theories and sub constructs, e.g., emotional intelligence (Simonet et al., 2021); interpersonal competence (Persich Durham and Robinson, 2022); social intelligence (Weis and Süß, 2005) and social skills (Dong et al., 2023)—thus needed to be narrowed down and Kanning (2002, pp. 154–163) theoretical model on social competence was applied as a reference theory. An analysis of existing intervention programs, and the potential need for further studies in the field as well as the specific problems, existing studies focused on, was conducted in a systematic review (Schüller and Demetriou, 2018, pp. 39–55).

Apart from that, a community needs assessment, targeted specifically toward the disseminating group of teachers, was conducted. A digital ranking tool was applied, which aimed at a ranking and prioritization of possible subcategories of social competences to create a study framework addressing the needs of the target implementation group (*N* = 35). To be able to identify relevant behavioral determinants and potential objectives, the questionnaire included 18 items, gathering definitions and rankings of aspects of social competences according to their importance, apart from further information on the sociometric data of teachers. It contained items for frame data on the schools of the teachers (three quantitative items), sociometric data on the teachers themselves (six quantitative items), definitions of aspects of social competence (four qualitative items) and ranking questions on the importance of these aspects (five ranking items) based on the theoretical model of (Kanning, 2002, pp. 154–163). The results were subsequently analyzed statistically as well as qualitatively using thematic analysis to create categories (Braun and Clarke, 2006, pp. 77–101; Braun and Clarke, 2019, pp. 589–597).

Based on the outcomes of quantitative and qualitative methods toward a sufficient needs assessment, a model of the problem was developed and the objectives of the intervention program (iii) were defined. Additionally, a community asset assessment was conducted to detect project partners and supporting structures (Supplementary Table S1).

#### 2.1.2 Application of step 2: logic model of change—program objectives and outcomes

The second step of IM aims toward a change of the problem identified afore (Bartholomew Eldrege et al., 2016). It includes desired outcomes and objectives and a selection of determinants and matrices of change objectives. Based on the results of the needs assessment and the aim of the intervention, a model of change is developed and change objectives are structured in change matrices. Desired outcomes are based on scientific findings on intervention effectiveness in studies resulting from research.

In a second step the gathered information was used to create an interview guide according to principles of the INVOLVE System (INVOLVE, 2012) with the aim to gain insights into the reasoning behind certain results of the teacher questionnaire and to focus on evaluability of the aims selected for the project. The INVOLVE System was developed by a research group focusing on the integration of stakeholders and public participation in health or social sciences. According to the approach, − based on the principles of the Equality Act (Equality and Human Rights Commission, 2010; Office for Disability Issues, 2011) – equal inclusion of teachers with more professional experience and teachers with rather limited professional experience was targeted. Additionally, an even distribution of male and female teachers was aspired to control for potential gender effects. Ensuring to offer an open interview setting with guideline questions preventing swayed or biased answers according to social desirability was prioritized. Interview guidelines included sociometric questions about the teachers (7 items on experience, age, subjects taught etc.), which corresponded with those asked in the teacher questionnaire and seven narrative impulses with corresponding category assignments as well as control and maintenance questions (e.g., “Do you remember situations in your own lesson planning or content, which targeted the social development of students?”). The items aimed to further explore the knowledge about social competence in theoretical and practical aspects, as well as the importance, methods and challenges of a promotion of social competences in a school setting, specifically in PE. Following up on that, the interviews were transcribed verbatim and answers were categorized in themes and subthemes (Lamnek, 2010).

The massive number of possible outcomes derived from the needs assessment, the teacher questionnaires, and the objective evaluation of method effectiveness in the systematic review were then narrowed down and validated toward usability and practicability using the qualitative input from the teacher interviews (e.g., the aspect extraversion—extent to which a person enjoys socializing—had to be excluded despite its effectiveness and the high ranking among teachers, because it describes a character trait, which cannot be promoted easily). The same method was applied to evaluate possible determinants and further extract the most important and changeable desired outcomes. The matrices included a cross-examination of relevant determinants in correlation with each of the objectives.

#### 2.1.3 Application of step 3: program design

The third step of IM focuses on the program design, including the generation of themes, scope and sequence planning. The selection of themes is conducted based on theory and evidence and then transferred into applicable lesson plans. Firstly, advantages and disadvantages of different methods (e.g., teacher-centered approaches vs. student-centered approaches) and their suitability were evaluated. The studies compared in the systematic review (Schüller and Demetriou, 2018, pp. 39–55) gave an overview of possible methods and applications and the effectiveness of different methods for the promotion of social competence was compared. The main criterion was effectiveness in different intervention fields. Thus, relying on Kanning (2002, pp. 154–163) theory, different methods for each of the three aspects (i.e., perceptive-cognitive, emotional-motivational, behavioral) were compared and selected using the knowledge of the teachers on educational determinants. Further steps included the development of methods that could be applied to different settings in PE (such as team sports and individual sports), their fit toward the general aim of the intervention and the specific aims derived from it and were illustrated in change matrices. The determinants under which these selected methods would work effectively were drawn from the systematic review results and the application knowledge of the teachers. A systematic of change objectives was matched with appropriate methods on both student and environmental level. Moreover, the planning included decisions regarding the target group of participants, the environmental level of the intervention and its duration, following the directions of the IM (Bartholomew Eldrege et al., 2016). Additionally, selections for a program theme, its components and sequence were generated following the guidelines, and a general construct of process evaluation measures was developed ensuring that it would fit the methods selected.

#### 2.1.4 Application of step 4: program production

After identifying possible methods and applications for specified program objectives included in the change matrices, the methods to be included in the program have to be arranged within the program structure (Bartholomew Eldrege et al., 2016). In the project the different levels of desired effects on the levels of perceptive-cognitive, emotional-motivational and behavioral aspects of social competences (Kanning, 2002, pp. 154–163) need to be matched with methods. The production of materials, including lesson plans and materials necessary for the lessons (such as apparatus plans etc.) follows afterwards.

#### 2.1.5 Application of step 5: program implementation plan

Step number 5 of IM focuses on the actual program implementation plan by considering context and setting of the intervention as well as potential users and time frames for the implementation (Bartholomew Eldrege et al., 2016). After that, outcomes and performance objectives were delivered in a teacher-training leaflet and implementers were instructed individually for 20–30 min in the intervention tools. The tools were also piloted with a group of students and teachers at a private school. Finally materials, scope, and themes were rearranged and changes that became visible during pretesting were included in the program prior to its application in the final intervention and control group.

#### 2.1.6 Application of step 6: evaluation plan

In step 6 of IM, an evaluation plan including effect and process evaluation questions is developed and the evaluation design is specified and tested. Therefore, the first step was to evaluate the measurement methods provided in the systematic review and compare them on different levels. These levels included an assessment of the instrument and the studies it was used in, the included variables, scales and number of items, and its validity and reliability. Moreover, it was evaluated whether the systems were easily available, accessible in the respective language and suitable for specific age groups.

## 3 Methods

### 3.1 Outcomes of step 1: needs assessment

Using the theoretical model of Kanning (2002, pp. 154–163) (Supplementary Table S2) the construct was distinguished in three main categories forming social competence: perceptive-cognitive, emotional-motivational and behavioral aspects, referencing three main aspects of the heuristic-systematic information processing model (Eagly et al., 1993) applied in social sciences.

As a result of the analysis of risk factors for low levels of social competence, it was concluded, based on the systematic review and the successful intervention programs depicted there, that future intervention programs should (a) promote social competence using rituals, role models and prompting (74.0% success rate) while focusing on (b) a reduction of inappropriate behaviors (85.0% success rate) and (c) including teacher trainings (62.1% success rate), apart from (d) addressing mainly adolescents (100% success rate vs. 66.7% success rate in children) and (e) ensuring that physical activity is promoted equally (Schüller and Demetriou, 2018, pp. 39–55). Theoretical background and study construct should be deduced from a conclusive line of intervention structure, including a clear definition and derivation from theoretical concepts. This resulted in the application of Kanning (2002, pp. 154–163) model of social competence, including a measurement tool (Kanning, 2009) derived from this model. Assessing the design of the studies included in the systematic review, it should focus on a high level of methodological quality and include multi-level assessment (e.g., self-assessment, observations by peers and family, network analysis and systematic observation by independent observers).

The results included a teacher preference for the four subcategories of social competences with their reflexive definitions by Kanning (2002, pp. 154–163): reflexibility with 91.66% of teachers as an umbrella construct for self-awareness and person perception (i.e., extent to which a person actively engages with themselves and their interaction partners), prosociality with 26.47% of teachers in the group of social orientation (i.e., extent to which a person actively engages with other people, helps them and acts in solidarity and fairness toward them), self-control with 17.65% (i.e., ability of a person to control his own behavior rationally even in stressful situations) and extraversion with 41.18% of teachers in the group of aggression aspects (i.e., extent to which a person likes to approach other people and make social contacts). Extraversion, also seen as an important aspect by the teachers, had to be excluded due to its localization in the character traits of children and adolescents (Goldberg, 1993, pp. 26–34), which are hard to manipulate.

Due to these needs assessment based results, the aim of the program was defined as: Students in the eighth grade of secondary schools participating in the six-month intervention will show changes in (a) realistic direct and indirect self-awareness; (b) increased person perception; (c) higher levels of pro-social behavior and self-control, and (d) an accurate representation of the self, compared to students who did not participate in the study. Finally, a logic model of the problem was created (Supplementary Figure S1) and the relevant determinants were rated along their importance (Supplementary Table S3).

### 3.2 Outcomes of step 2: program objectives and outcomes

Program Objectives and Outcomes (Table 1; Supplementary Table S4) were informed by two levels of information with one being the student level and the second being the level of environmentally involved stakeholders (such as teachers). Thus, focusing on the levels of the development of social competences: situation analysis, the analysis of behavioral options, the application and evaluation of behaviors (Kanning, 2002, pp. 154–163) on a student level and considering interpersonal and community assets on an environmental level.

**Table 1:** Behavioral and environmental outcomes.

Level	Generalized Aim	Description
Student level	Situation analysis	Students develop appropriate and various skills to be able to analyze diverse situations requiring social competent behavior. They acquire means to assess their direct and indirect self-awareness and person perception.
	Analysis of behavioral options	Students consider alternative reactions toward behaviors and try to anticipate consequences.
	Application of behaviors	Students apply behaviors in different contexts, consider, and deal with feedback appropriately.
	Evaluation	Students integrate evaluation results and feedback in their social repertoire.
Environmental level	Interpersonal	Fellow-students, (relatives and friends) offer space to apply and test behaviors in different situations and with various opponents and encourage it.
	Organizational	Teachers offer possibilities to apply appropriate behaviors with various interaction partners and include them in the classroom management. They demand and promote pro-social behavior.
	Community	Teachers help to develop and defend social values and standards.

**Figure 1:**
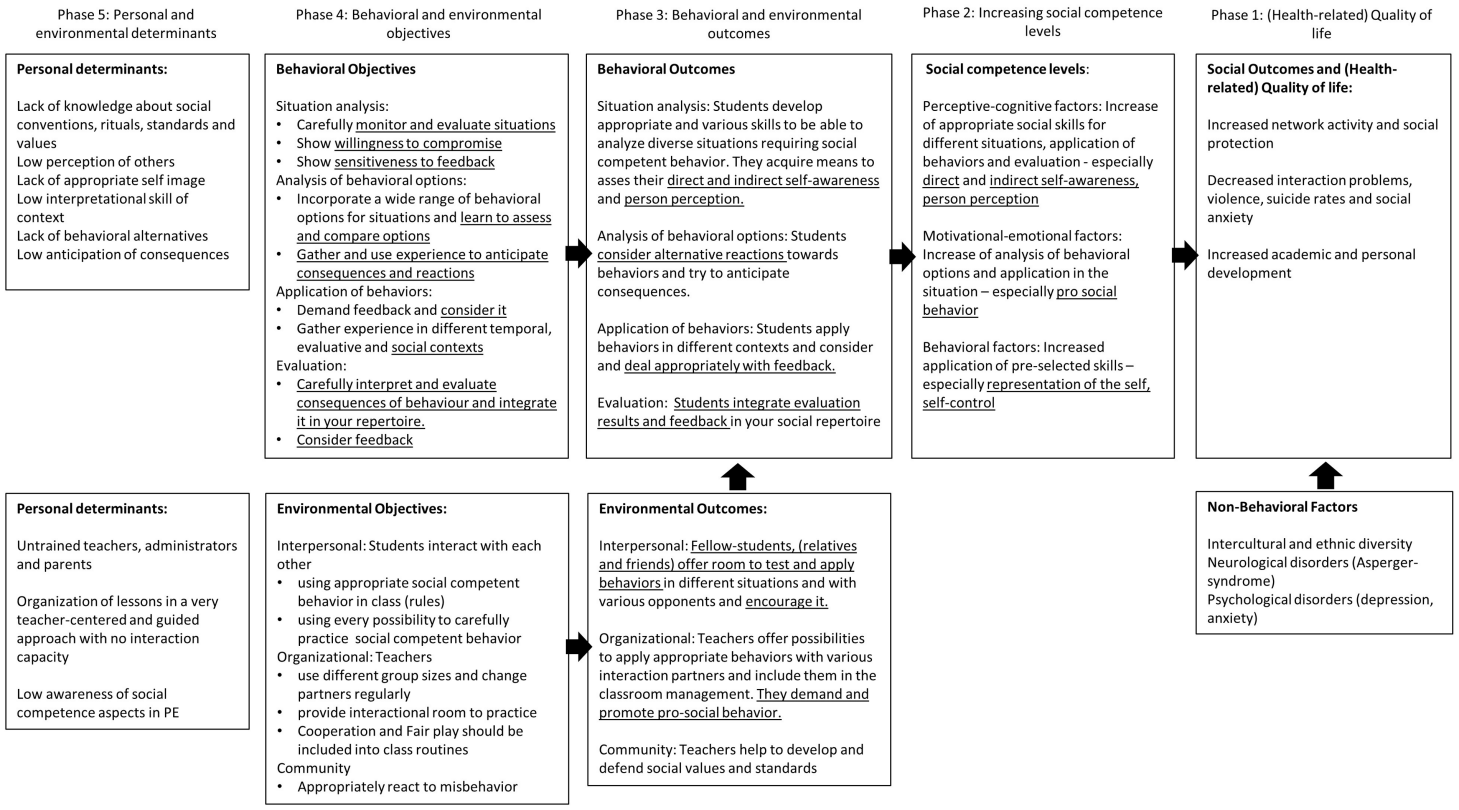
Model of change.

The logic model of the problem was then adapted and evolved toward a model of change, stating specific pathways to achieve program goals and effects (Figure 1). It was supplemented by specific determinants and change objectives, which were combined according to their common, generalized terms and consolidated in a matrix for each of the objectives (Table 2).

**Table 2:** Matrix of performance analysis and personal determinants for the situation analysis component of social competences.

Situation analysis: Students develop appropriate and various skills to be able to analyze diverse situations requiring social competent behavior. They acquire means to assess their direct and indirect self-awareness and person perception						
Performance objectives (Students)	Personal determinants					
	Knowledge: Lack of knowledge about social conventions, rituals, standards and values	Perception: Low perception of others	Self-image: Lack of appropriate self-image	Context-Interpretation: Low interpretational skill of context	Behavior: Lack of behavioral alternatives	Consequence awareness: Low anticipation of consequences
PO1: Increase careful evaluation of situations	K.1a. Recall different situations and their consequences K.1b. Practice evaluation skills (taking a step back, knowing about social conventions etc.)	P.1. Increase attention toward others and their actions	S.1a. Assess image of the self appropriately S.1b. Be sensitive to reactions of others	I.1a. Distinguish similar contexts I.1b. Practice interpretation skills I.1c. Analyze situations correctly	_	C.1. Increase attention toward possible consequences
PO2: Show willingness of compromise	K.2a. Recognize necessity of compromising K.2b. Be emotionally stable in order to deal with having to compromise	P.2a. Interpret behavior of others generously P.2b. Accept/evaluate ideas of others	S.2a. Be open to different views of your personality	I.2. Accept different interpretations of the context	_	C.2. Apply compromise means in order to avoid consequences
PO3: Show sensitiveness to feedback	K.3a. Identify feedback on standard situations K.3b. Adapt behavior accordingly	P.3.Identify valuable input	S.3. Adapt self-image	I.3. Evaluate different interpretations of the context	_	C.3. Value feedback on possible consequences

### 3.3 Outcomes of step 3: project design

**Figure 2:**
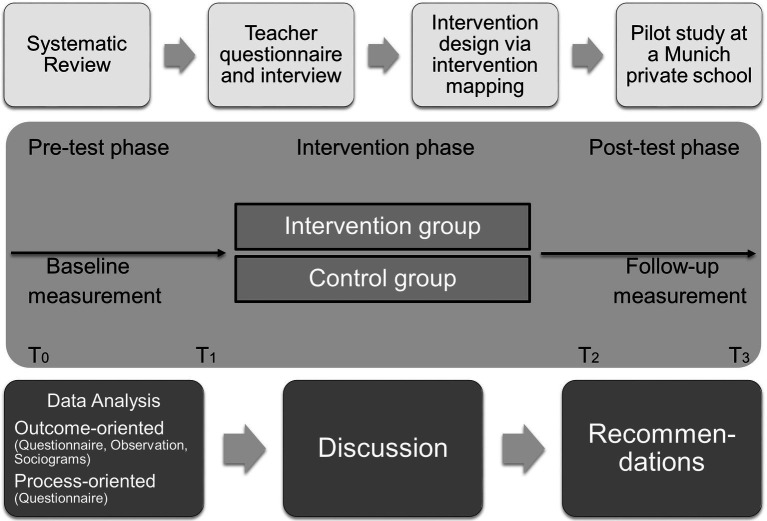
Project scope and design.

First, the effectiveness of methods was compared in 28 selected articles found in the systematic review (Schüller and Demetriou, 2018, pp. 39–55), resulting in main method groups that were relevant. Thus, a teacher training was developed, including instructions on the reinforcement of positive examples of social competent behavior in students (Fryling et al., 2011). Finally the general scope of the project design (Figure 2) was discussed and finalized with pre-intervention steps (i.e., intervention design and pilot study phase), three testing phases and three post-test-phase steps.

### 3.4 Outcomes of step 4: program production

**Figure 3:**
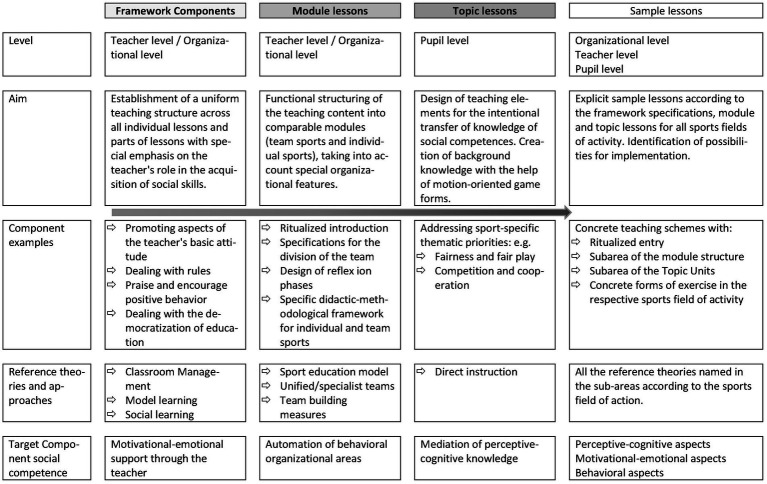
Theoretical construct of intervention content adapted from Schüller and Demetriou (2019, pp. 548–552).

A general model for the intervention system was constructed including different levels of intervention methods (Figure 3). An intervention program with different lesson types was created. It included a general frame with a rule contract, basic rules that needed to be followed and were controlled and adapted by the teacher, and prompt and praise (Bozkuş, 2021). The theoretical input used, was model learning (Fryling et al., 2011) and methods of classroom management (Bozkuş, 2021), which showed great results in the promotion of positive behavior (Dohrn et al., 2001), self-control and problem solving skills (Androjna et al., 2000, pp. 1–6) in students. Each lesson was framed by a self-chosen ritual and ended with a team-building challenge (Lazarides et al., 2024). On the student level, three different lesson levels were applied, including topic lessons which intentionally taught knowledge on social competence such as fairness and fair play (Schüller, 2019a, pp. 557–560) as well as cooperation and competition (Schüller, 2019b, pp. 553–556). On a second level, the lessons were divided into team sports and individual sports because the two demanded different aspects of social competence and the so called “module lessons” were based on either the sport education model (Hastie and Wallhead, 2023) in team sports or cooperative learning (Beals et al., 2024) in individual sports, which have shown to be effective in positively influencing aggression and passiveness (García-López and Gutiérrez, 2015, pp. 1–16). Specific model lessons were then constructed as example lessons including the frame aspects and topic/module lessons.

In the next step, the lessons were adapted toward the curriculum also including the gender separation aspects relevant in this state. The lessons included the main topics for eight grade and generalized some of the distinctive sports field such as basketball and handball, which were followed in lessons for more generalized aspects of ball games, including dribbling and aim throws. Every piece of material needed for the lessons that were part of the intervention was constructed based on methodical and didactical literature for teachers and adapted using the theoretical background behind each lesson level and the methods picked. The first drafts of the program were then tested with a group of teachers and students for applicability and usability. After pre-testing and piloting with two school classes of a slightly lower age, it was determined whether materials were age appropriate and easily applicable for teachers. This was followed by the construction of an implementation plan, including possible methods and applications and a general implementation plan with possible stakeholders in the next step. Finally, a dissemination plan for different intervention groups was designed.

### 3.5 Outcomes of step 5: program implementation plan

Intervention planning was accordingly followed by the adaptation of the program toward the needs of the target groups of implementers and students. Within the program development a total of 14 lessons on different fields of exercise and sports were produced (three on gymnastics—esp. acrobatics; three on athletics—esp. track and field; two on dancing; two on ball games—esp. handball and basketball; two on soccer; two on setback games—esp. volleyball), based on the requirements of the curriculum and the age-group. Additionally, two topic entities on fairness and cooperation, and competition were constructed and could be added to any of the exercise related lessons. The exercise fields of winter sports and swimming were left out, due to special requirements concerning materials and space, which could not have been controlled in the intervention. Some of the lessons were adapted to be applicable in- and outdoors. A specific teacher manual with relevant aspects of implementation rules, a manual on how to provide feedback within the lessons and how to indicate, which parts had been implemented, was constructed.

Adaptation toward the target group of students was based on findings of developmental sensitivity during adolescence (see introduction). It was decided that the first implementation should include participants with non-specific needs to assess the program effectiveness prior to adapting it toward specific needs groups (e.g., classes with a history of bullying and aggression or student groups with a physical or psychological precondition).

Concerning the environmental level, it was established that the intervention should be implemented on the school level in structurally organized PE lessons to guarantee a controlled surrounding before eventually expanding the implementation into after-school programs, classroom teaching and the homes of students. The duration of the intervention period had to be kept flexible due to its nature of field research, subsequently including a period of 4 months in order to ensure that snow sports weeks, absence of teachers etc. would still allow for the necessary amount of intervention lessons to be implemented.

Apart from the fact, that one team sport and one individual sport needed to be covered within the implementation, the teachers were free to choose from the lessons. Every teacher received a folder with all the material necessary and was schooled accordingly within 20–30 min by the same instructor.

### 3.6 Outcomes of step 6: evaluation plan

Based on the information gathered from the comparison of study designs and measurement instruments within the systematic review (Schüller and Demetriou, 2018, pp. 39–55) multiple levels were included in the evaluation plan (Supplemental Figure S2) and the number of test dates was determined. The evaluation plan was further adapted to the theoretical base of the intervention plan and focused on the findings and the theoretical construct of Kanning (2002, pp. 154–163) with its three clusters of social competence aspects (i.e., perceptive-cognitive, emotional-motivational, behavioral). Moreover, different implementation levels were evaluated including students in the intervention and control group as well as PE teachers. It was necessary to include both, the effect and the program and process evaluation aspects. Using a mixed-methods approach was considered beneficial in order to assess the different levels of social competent behavior (situation analysis, analysis of behavioral options, the application and evaluation of behaviors (Kanning, 2002, pp. 154–163)).

Therefore, the effect evaluation consists of a student questionnaire on sociodemographic aspects from the MOMO study (Schmidt et al., 2016), knowledge aspects of social competence - including qualitative and quantitative items -, questions derived from of Kanning’s three-dimensional evaluation tool (Kanning, 2009) and a social network analysis (Wasserman and Faust, 2007). Additionally, items on the program evaluation are added, including students perception of the teacher behavior, perceivable changes in lesson content and structure, and short scales of Kanning’s multi-dimension tool (Kanning, 2009). Finally, each evaluation date on pre-test, pre-test 2, post-test and follow-up includes a behavior observation by independent observers in a normative setting. The first tool is designed and pretested specifically for the study and an additional standardized pre-existing observation tool SOCARP by Ridgers et al. (2010, pp. 17–25) in a free lesson atmosphere is added. Both observation tools measure aspects of social competences, while also including items on physical activity levels. It was further decided to have each teacher assess the changes in each of the students’ behavior on a subjective level.

Process evaluation takes place throughout the intervention phase and is conducted at two evaluation dates. It consists of a short questionnaire with items on the perceived study process on a student’s level and items in a teacher questionnaire on the study design and the application of program contents. Teachers also evaluate the lessons, and their social competence levels are evaluated similarly to those of the students via questionnaire, as only a teacher whose own competence is adequate can translate the necessity of social competence onto the student’s level (Fryling et al., 2011). Lastly, teachers also give feedback on the lessons and how they adhered to the lesson plans.

The evaluation of data is planned to refer back to the theoretical construct and include data of the different methods applied to each of the three aspect groups (perceptive-cognitive, emotional-motivational, behavioral), thus resulting in an evaluation that includes multiple perspectives for each. Perceptive-cognitive aspects therefore include questionnaire data, qualitative input on how students defined social competence and teacher evaluations of student’s self-awareness. Emotional-motivational aspects include questionnaire data and teacher evaluations of self-control, while behavioral aspects will be evaluated through questionnaire data, teacher evaluations of student’s solidarity, peer assessment data on success- and emotion-oriented rankings and observational data from the research team. Using this data it will be possible to assess each of the three dimensions and calculate a construct score for each, as well as for the overall construct of social competences for each student on each time-point. With the help of these construct scores and sociodemographic influences unique to each student it will be possible to analyze the individual student’s position in the social networks and the influences this data might have on the roles depicted in the networks, while taking into account the three different time point.

## 4 Discussion

The application of IM as depicted in the process described afore, aims at the systematic derivation, development and implementation of (a) a logically derived theoretical background, (b) evidence-based program objectives, (c) effective intervention methods, and (d) assessment tools and strategies that fit the theoretical model upon which the intervention on promoting social competence in PE is based. Although IM has been used to develop interventions for sociological and psychological outcomes (Leerlooijer et al., 2014, pp. 598–610), applying it to a behavioral process as an outcome is a novel approach requiring further examination.

### 4.1 Discussion of the IM steps

The first step of the IM process—the logic model of the problem and the needs assessment—was challenging due to the fact, that a behavioral approach was applied to shape behavioral processes through health-related content. Although social competences are evidently a main factor of health related quality of life, they are associated with mental wellbeing rather than with physical comfort (Holopainen et al., 2012, pp. 199–212; World Health Organization, 2007). Due to this, the model of the problem needed to be targeted toward internal perceptive-cognitive and emotional-motivational aspects of behavior development, as well as external behavioral outcomes (Kanning, 2002, pp. 154–163). Apart from that, the interactional component of social competence with intrapersonal and interpersonal components also needed to be considered (Kanning, 2002, pp. 154–163).

Developing interpersonal and community assets on an environmental level in step two of IM – the logic model of change and identification of program objectives and outcomes –, it became clear that, apart from offering judgment free and open feedback on behaviors (Taborsky and Oliveira, 2012, pp. 679–688), the determinants and objectives on that level were rather limited, if a controlled intervention setting was required. The model of change also supported this finding by showing that the organizational, interpersonal and community objectives and outcomes were opening up opportunities and a safe space for testing social behavior. It became obvious, that this created room for different social contexts and interactions, but would be challenging in terms of the evaluation of the impact of environmental objectives.

The project design (step 3 of IM) was created very effectively due to the various results of the teacher questionnaire and the insights gathered through the systematic review. However, due to the aim of promoting social competences, it was necessary to evaluate which target group of students would respond most effectively to an intervention program. Thus, it became relevant to not only discuss target groups with a risk for low levels of social competences, but also especially include participants in a developmentally sensitive age group. Establishing that, it was decided to focus on the target group in which social competences are developed. Future applications include a follow-up study targeting high-risk groups with specific needs, such as participants with a history in bullying or aggression (Bull et al., 2009, pp. 312–317) and physically or cognitively impaired students (e.g., with Asperger’s syndrome) (Andanson et al., 2011, pp. 589–596). Additionally, interventions transferring social competences into other subjects and areas of life are planned.

Within the program production (step 4 of IM) and the program implementation plan (step 5 of IM) it was possible to adapt the program design to the specific needs not only of the needs group, but also to the curricular framework of the schools. Nevertheless, it was not possible to apply the intervention content to every curricular topic, due to organizational restrictions (e.g., no swimming lessons planned for the term). Moreover, teacher instruction of the intervention contents showed that especially the nature of social competences as a rather abstract construct needed to be thoroughly discussed to ensure that the implementers - teachers in this case - were able to convey the contents accordingly.

The construction of an evaluation plan (step 6 of IM) showed that different levels of intervention also required various methods of evaluation. Apart from that, it became relevant to have the teachers as implementers evaluated in their own believes as well, as they would serve as behavioral models (Fryling et al., 2011). Furthermore, the systematic derivation of the theoretical model showed that, they not only needed to be evaluated in their own believes and levels of social competences but also in their ability to transfer their knowledge and intentions on a student level on a communicational level (see: mathematical theory of communication by Shannon and Weaver, 1963). The margin of error in this area—on a level of teacher education, on a level of teaching abilities and on a level of student recipient abilities—was shown to be not controllable otherwise. Having discussed this in advance, it was possible to include multiple evaluation methods on different levels such as a questionnaire on the teachers levels of social competences, the students perception of the teachers intentions and the student level of social competences itself.

### 4.2 Limitations and chances of the IM approach in promoting social competences

The limitations of IM in educational settings mainly lie in the fact, that it was not constructed for this specific intervention content, but was originally implemented for risk reduction (van Empelen et al., 2003, pp. 402–412), health promotion and screening (Fernández et al., 2005, pp. 394–404), early detection or adherence and self-management interventions (Detaille et al., 2010). The promotion of social outcomes through behavioral change methods (Bartholomew Eldrege et al., 2016), increases the risk of not clearly distinguishing outcomes and aims from methods and mediators within the mapping process. Nevertheless, the application of a systematic intervention planning approach toward sociological outcomes is a first innovative attempt to methodologically structure and derive intervention content and study design. It offers numerous opportunities to further develop and establish a systematic approach of intervention and study designs in the educational settings.

Apart from that, the environmental and community asset in this setting not only functions as a determining factor, but also as an interactional context und influencing factor, meaning that the interpersonal aspect is part of the learning progress, since peer and teacher feedback mainly shapes the evaluation process of behavior and cannot be seen as separate from the individuals progress on social competences. This becomes specifically obvious in the temporal, social and evaluative reference points of behavior, which determine whether a behavior is suitable in a specific situation (temporal), with a specific interaction partner (social) and with a desired evaluative outcome (positive evaluation of behavior) (Kanning, 2002, pp. 154–163). This limitation once more emphasizes the need for education specific IM approaches, that address the needs of program planning in this settings more precisely.

Moreover, interventions on interaction aspects should not only focus on one specific setting, but rather include as many interactional contexts as possible, such as family, friend groups outside of the school settings etc., in order to generalize behaviors (Blazevic, 2016). Although this dimension of social competence was clear from the beginning, it was not possible to include these into the study because a different testing strategy would have been needed in order to control the various variables. A future application should therefore aim toward a more diversified settings approach and also target transferable skills, diversifying the IM approach toward an implementation specific approach and tailoring the program toward more interactional settings.

Furthermore, the evaluation of social competences was found to be challenging, as the intrapersonal aspects of situation analysis and the analysis of behavioral options are processes within the individual, which can only be evaluated through subjective reports and answers of the participant. Moreover, the recipient in the interaction also contributes to this progress by their own intrapersonal evaluation of the behavior (Kanning, 2002, pp. 154–163), which leaves the objectively perceivable social behavior as a rather small part of socially competent interactions (Rose-Krasnor, 1997, pp. 111–135). Multi-method assessments, such as mentioned above offer a sufficient possibility to target the need for multi-level assessments in complex outcome constructs. Within the area of social competences it is also necessary to not only generate generalized definitions of the construct (Rose-Krasnor, 1997, pp. 111–135), but also include novel and innovative evaluation approaches such as network analyses; biomarkers of social interactions analyzing gaze behavior; facial expressions or voice characteristics (Drimalla et al., 2020).

Finally, the project showed rather low stakeholder involvement after the stage of the needs assessment and the definition of program outcomes and objectives, since a scoping review on the effectiveness of stakeholder engagement following the IM in a health care setting found that it is unclear whether stakeholder involvement in IM and the application of IM itself is positively associated with an effective promotion of the intervention objectives (Majid et al., 2018). Although the risk of stakeholders especially focusing on contributing socially acceptable aspects of the process und thus having a social desirability bias in the program design might have been given (Larson, 2018), in hindsight this would needed to be changed in order to increase the acceptability of the program within the target group (Bartholomew Eldrege et al., 2016; Jagosh et al., 2012).

## 5 Conclusion

This study set out to develop a systematically derived, evidence-based intervention program for promoting social competence in Physical Education (PE) using the Intervention Mapping (IM) approach. Our research has demonstrated that IM is a valuable tool for addressing the complex, interactional nature of social competence development within educational settings. Within the project, IM was especially relevant as it ensured that specific challenges such as the interactional nature of the content and the outcomes could be addressed early on. The pilot phase of the project has in the meantime been completed and is currently in the evaluation stage. Its assessment of various types of intervention content, program applications and evaluation methods are offering valuable insights for an application toward a larger participant group and the adaptation toward specific needs groups, such as those with psychological impairments or a known record in abuse and bullying. Moreover, an advancement toward applications within all social contexts of social competence is possible.

The application of IM in this context has yielded several key insights: First, theory-based planning can be improved and is crucial when addressing complex constructs, such as social competence. Using the IM process as a guidance in the form of the IM approach (Bartholomew Eldrege et al., 2016), the Matrix Assisting Practitioner’s Intervention Planning Tool (O'Cathain et al., 2019) or the Precede-Proceed Model (Lusk, 1992) offers opportunities to systematically plan, develop and evaluate intervention programs within different contexts.

Secondly, the pilot study showed potential toward the adaptation of the intervention for various contexts and target groups, including those with specific needs such as psychological impairments or histories of bullying, which is essential for effectively engaging diverse target groups. This aligns with findings from research, showing that IM recently gained traction as a model for implementation mapping, which might be interesting for the further development and application of IM in educational settings. Within this approach, IM is adapted to be a process applied to research that does not aim at the development of a new intervention program, but focuses on the targeted implementation of an existing intervention using implementation science. It is thus also based on the steps of the IM, but relates to a specific target and implementation group during every step of the protocol, aiming at addressing target-group specific barriers and facilitators, as well as developing strategies (Fernandez et al., 2019a, p. 209).

Further developments in the field of intervention planning in an educational setting should include the multi-method assessment approaches considering novel ideas, such as biomarker assessments, which ensure that complex constructs such as social competence can be assessed more thoroughly and a higher stakeholder involvement. By addressing these areas, future interventions are able promote social competence in PE more effectively, potentially leading to improved social, emotional, and academic outcomes for students. This research thus provides a foundation for more targeted, effective approaches to fostering crucial social skills within educational environments.

## Data Availability

The original contributions presented in the study are included in the article/Supplementary material, further inquiries can be directed to the corresponding author.
