# Supplementary material for: Strengthening social skills: developing a social competence intervention for physical education using intervention mapping—protocol paper
Source: Front Psychol. 2025 Feb 18;16:1484943. doi: 10.3389/fpsyg.2025.1484943 (PMC11876426; doi:10.3389/fpsyg.2025.1484943)

Supplementary Material

# Supplementary Data

## Supplementary Tables

**Supplemental Table S1**

*Community Assets Assessment*

| **Social Environment Asset Assessment** | **Information Environment Asset Assessment** | **Policy/Practice Environment Asset Assessment** | **Physical Environment Asset Assessment** |
| --- | --- | --- | --- |
| Existing social environment factors that could support the project:   1. Teacher trainings 2. Student-centered education | Existing communication channels usable for the program:   1. Facebook 2. Twitter 3. Instagram 4. Local radio 5. Newspapers 6. School bulletins | Existing policies and practices to support the program:   1. DFG, BMBF 2. Educational Ministry 3. Cities of project execution 4. School project teams 5. Parent and family support | Aspects of natural or built environment:  none |
| Existing organizations to support the project:   1. Teacher organizations 2. Health Insurances |  |  |  |

**Supplemental Table S2**

*Theoretical Construct of Social Competences, based on Kanning (2009)*

| Social Competences | | |
| --- | --- | --- |
| Perceptive-cognitive aspects | Emotional-motivational aspects | Behavioral aspects |
| - Self-awareness - Perception of others - Perspective-taking - Locus of control - Decisiveness - Knowledge | - Emotional stability - Pro-sociality - Value diversity | - Extraversion - Assertiveness - Flexibility in action - Communication skills - Conflict behavior - Self-control |

**Supplemental Table S3**

*Rating of importance and changeability of the determinants*

| Determinant | Importance | Changeability | Evidence for importance |
| --- | --- | --- | --- |
| Personal Determinants | | | |
| Knowledge: Lack of knowledge about social conventions, rituals, standards and values | ++ | +++ | Successful promotion of perceptive-cognitive aspects in 75% of studies (Schüller & Demetriou, 2018) |
| Perception: Low perception of others | ++ | + |  |
| Self-image: Lack of appropriate self-image | + | + | Successful promotion of emotional-motivational aspects in just 14,3 % of studies (Schüller & Demetriou, 2018) |
| Context-Interpretation: Low interpretational skill of context | +++ | ++ |  |
| Behavioral Alternatives: Lack of behavioral alternatives | +++ | +++ | Successful promotion of behavioral aspects in 85% of studies (Schüller & Demetriou, 2018) |
| Consequence awareness: Low anticipation of consequences | + | ++ |  |
| Environmental Determinants | | | |
| Untrained teachers, administrators and parents | +++ | +++ | Successful promotion of social competences in 62,1 % of studies with teacher training (Schüller & Demetriou, 2018) |
| Organization of lessons in a very teacher-centered and guided approach with no interaction capacity | ++ | + | Successful promotion of social competences in 62.6% of the studies with instructional approaches and 76.9% in studies staging of situations requiring social competent behaviors (Schüller & Demetriou, 2018) |
| Low awareness of social competence aspects in PE | +++ | ++ |  |

Note: Importance = the strength of the evidence for the causal relationship between the determinant and the behavior that should be changed; changeability = the strength of the evidence that the proposed change can be realized by the intervention; + = NOT very important/easy to change; ++ = important, changeable; +++ = VERY important/changeable

**Supplemental Table S4**

*Outcomes and Performance Objectives*

| **Behavioral Outcomes and Objectives** |
| --- |
| 1. **Situation analysis:** Students develop appropriate and various skills to be able to analyze diverse situations requiring social competent behavior. They acquire means to assess their direct and indirect self-awareness and person perception. Students will… |
| - 1. increase careful evaluation of situations.   2. show willingness of compromise.   3. show sensitiveness to feedback. |
| 1. **Analysis of behavioral options**: Students consider alternative reactions towards behaviors and try to anticipate consequences. Students will… |
| - 1. incorporate a wide range of behavioral options for situations and learn to assess   and compare options.   - 1. gather and use experience to anticipate consequences and reactions. |
| 1. **Application of behaviors:** Students apply behaviors in different contexts and consider and deal appropriately with feedback. Students will… |
| - 1. demand feedback.   2. gather experience in different temporal, evaluative and social contexts. |
| 1. **Evaluation:** Students integrate evaluation results and feedback in your social repertoire. Students will… |
| - 1. carefully interpret and evaluate consequences of behavior and integrate it in your   repertoire.   - 1. consider feedback. |
| **Environmental Outcomes and Objectives** |
| 1. **Interpersonal:** Fellow-students, (relatives and friends) offer room to test and apply behaviors in different situations and with various opponents and encourage it. Fellow students will… |
| - 1. use appropriate social competent behavior in class (rules).   2. use every possibility to carefully practice social competent behavior. |
| 1. **Organizational:** Teachers offer possibilities to apply appropriate behaviors with various interaction partners and include them in the classroom management. They demand and promote pro-social behavior. PE-teachers will… |
| - 1. change interaction partners (in class) regularly.   2. provide interactional room to practice.   3. include cooperation and fair play into class routines. |
| 1. **Community:** Teachers help to develop and defend social values and standards. Teachers will… |
| - 1. appropriately react to misbehavior |

## Supplementary Figures

**Supplemental Figure S1**

*Logic Model of the problem*


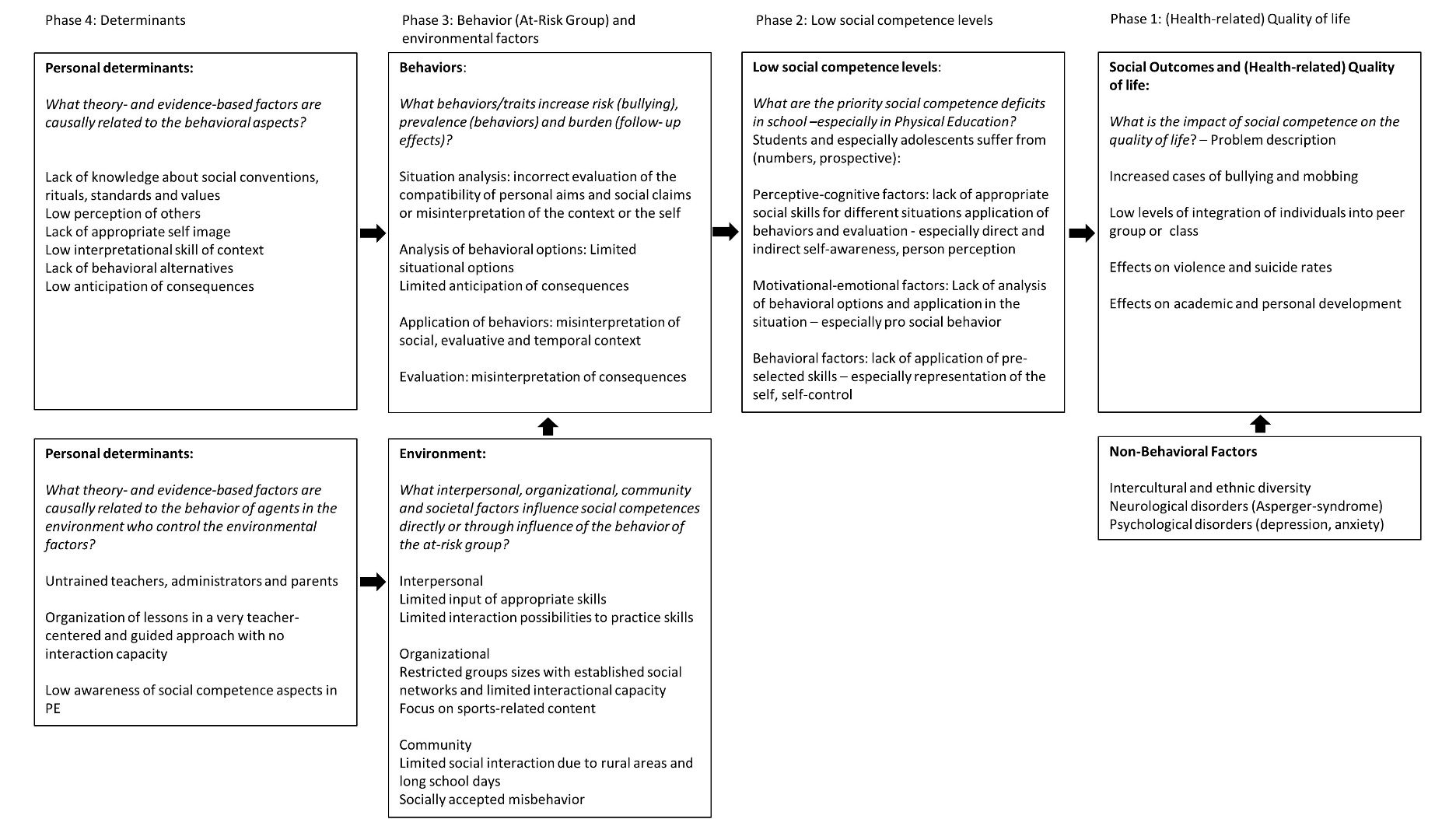


**Supplemental Figure S2**

*Evaluation Design*


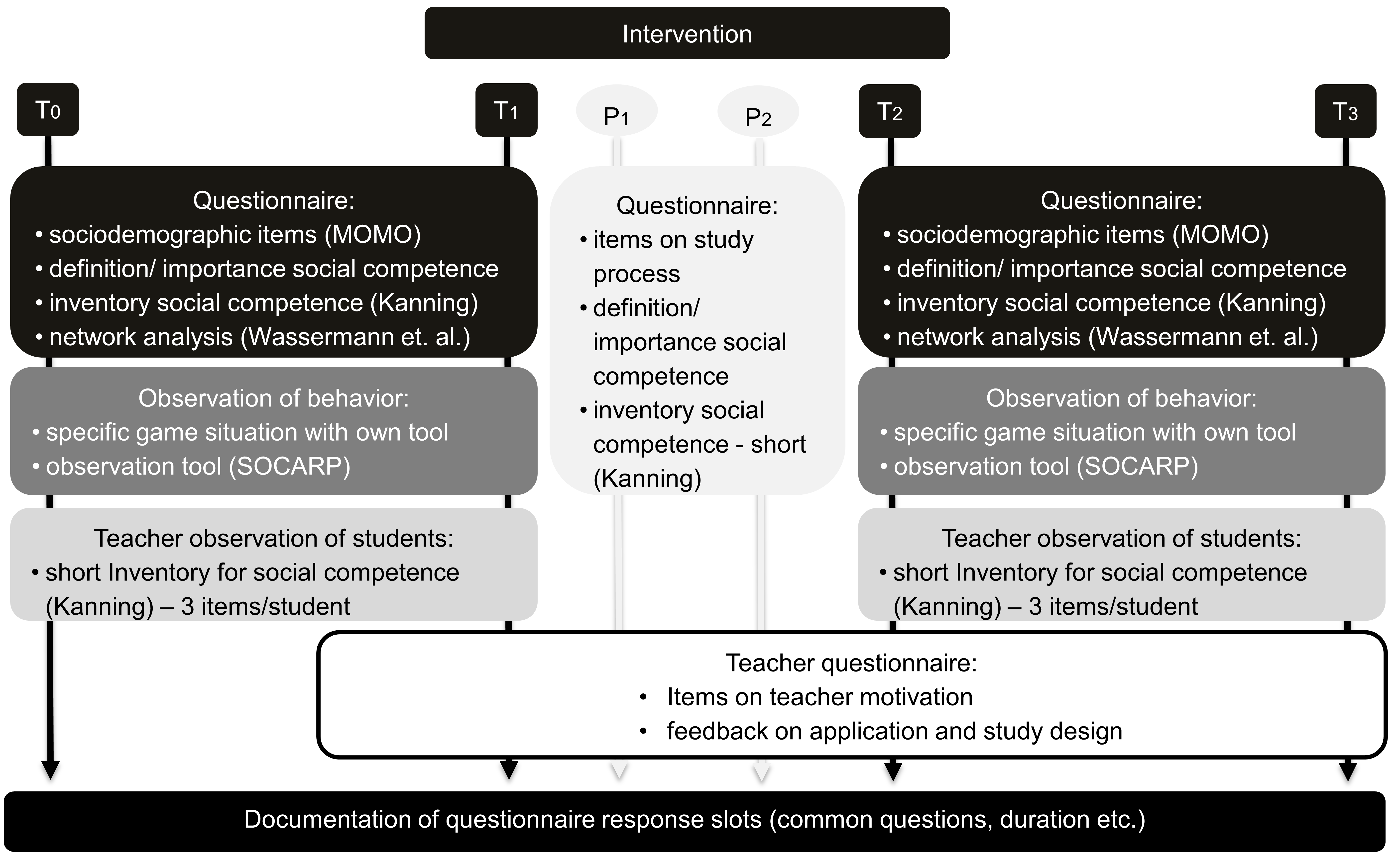

Supplement: Supplementary file 1 [file Data_Sheet_1.docx]
